# Artificial Intelligence Education Programs for Health Care Professionals: Scoping Review

**DOI:** 10.2196/31043

**Published:** 2021-12-13

**Authors:** Rebecca Charow, Tharshini Jeyakumar, Sarah Younus, Elham Dolatabadi, Mohammad Salhia, Dalia Al-Mouaswas, Melanie Anderson, Sarmini Balakumar, Megan Clare, Azra Dhalla, Caitlin Gillan, Shabnam Haghzare, Ethan Jackson, Nadim Lalani, Jane Mattson, Wanda Peteanu, Tim Tripp, Jacqueline Waldorf, Spencer Williams, Walter Tavares, David Wiljer

**Affiliations:** 1 Institute of Health Policy, Management and Evaluation Dalla Lana School of Public Health University of Toronto Toronto, ON Canada; 2 University Health Network Toronto, ON Canada; 3 Vector Institute Toronto, ON Canada; 4 Michener Institute of Education University Health Network Toronto, ON Canada; 5 Faculty of Medicine University of Toronto Toronto, ON Canada; 6 Institute of Biomedical Engineering University of Toronto Toronto, ON Canada; 7 Wilson Centre Toronto, ON Canada; 8 CAMH Education Centre for Addictions and Mental Health (CAMH) Toronto, ON Canada

**Keywords:** machine learning, deep learning, health care providers, education, learning, patient care

## Abstract

**Background:**

As the adoption of artificial intelligence (AI) in health care increases, it will become increasingly crucial to involve health care professionals (HCPs) in developing, validating, and implementing AI-enabled technologies. However, because of a lack of AI literacy, most HCPs are not adequately prepared for this revolution. This is a significant barrier to adopting and implementing AI that will affect patients. In addition, the limited existing AI education programs face barriers to development and implementation at various levels of medical education.

**Objective:**

With a view to informing future AI education programs for HCPs, this scoping review aims to provide an overview of the types of current or past AI education programs that pertains to the programs’ curricular content, modes of delivery, critical implementation factors for education delivery, and outcomes used to assess the programs’ effectiveness.

**Methods:**

After the creation of a search strategy and keyword searches, a 2-stage screening process was conducted by 2 independent reviewers to determine study eligibility. When consensus was not reached, the conflict was resolved by consulting a third reviewer. This process consisted of a title and abstract scan and a full-text review. The articles were included if they discussed an actual training program or educational intervention, or a potential training program or educational intervention and the desired content to be covered, focused on AI, and were designed or intended for HCPs (at any stage of their career).

**Results:**

Of the 10,094 unique citations scanned, 41 (0.41%) studies relevant to our eligibility criteria were identified. Among the 41 included studies, 10 (24%) described 13 unique programs and 31 (76%) discussed recommended curricular content. The curricular content of the unique programs ranged from AI use, AI interpretation, and cultivating skills to explain results derived from AI algorithms. The curricular topics were categorized into three main domains: cognitive, psychomotor, and affective.

**Conclusions:**

This review provides an overview of the current landscape of AI in medical education and highlights the skills and competencies required by HCPs to effectively use AI in enhancing the quality of care and optimizing patient outcomes. Future education efforts should focus on the development of regulatory strategies, a multidisciplinary approach to curriculum redesign, a competency-based curriculum, and patient-clinician interaction.

## Introduction

### Background

The widespread and rapid adoption of artificial intelligence (AI) technologies in health sciences, education, and practices introduces new ways of delivering patient care [1]. AI encompasses a broader term within computer science, which includes technologies that can incorporate human-like perception, intelligence, and problem-solving into complex machines [2]. Big data in health care, along with high-performance computing power, has enabled the use of AI, machine learning (ML), and deep learning, in particular, to improve clinical decision-making and health sector efficiency [3]. More recently, AI-enabled technologies have continued to emerge, predominantly in the medical fields of radiology, anesthesiology, dermatology, surgery, and pharmacy [4-7]. Although AI is not likely to replace clinical reasoning, Mesko [8] predicts that AI will influence all specialties in varying degrees, depending on the nature of the practice (eg, the degree of repetitive tasks involved and whether the tasks are data driven). However, the efficacy of AI-enabled technologies in health care depends on the involvement of health care professionals (HCPs) in developing and validating these technologies. Therefore, HCPs should play a role in this transformation and be involved in every aspect of shaping how AI adoption will affect their specialties and organizations.

Recommendations for HCP involvement are emerging. For instance, in the field of medical imaging, West and Allen [9] recommend that HCPs be involved in (1) implementing data standards and following them in practice, (2) prioritizing use cases of AI in medicine, (3) determining the clinical impact of potential algorithms, (4) describing and articulating the needs of the profession for data scientists and researchers, and (5) participating in the translation of practice needs from human language into machine language. As these technologies emerge, it is essential for HCPs and educators to have the competencies required to rapidly develop and incorporate these changes into their practices and disciplines.

At the individual level, a lack of AI literacy is a significant barrier to the adoption and use of AI-enabled technologies to their full capacity in various medical specialties. In AI education programs specifically, there are barriers to implementation at various levels of medical education (undergraduate, postgraduate, practice-based education, or continuing professional development). For instance, health informatics plays a valuable role in modern medicine; yet, it is not the focus of most medical school curricula [2]. Technology experts are often consulted to provide training on the use of electronic clinical tools, but this does not support the level of skill required to understand how it could be used to enhance patient interactions and improve care [10]. Another example exists within radiology residency programs, where the lack of awareness as well as lack of knowledge of implementing and using AI were cited as barriers to its adoption [11,12]. Incorporating AI fundamentals into health professionals’ curricula is essential, and it would be useful to balance this knowledge with providing patient-centered care by empowering future HCPs to consider AI in the context of their own clinical judgment. The combination of trust in their own judgment and basic statistical knowledge will be useful in understanding how to best apply new AI-driven technologies in clinical practice [13]. AI needs to be considered within the context of HCPs’ broader skill sets, priorities, and ultimate goals in health care; this includes encouraging patient-centered, compassionate care in clinical practice [13,14].

Martec’s Law refers to the idea that technology changes occur much more rapidly, and in fact exponentially, compared with the ability of organizations to adopt these technologies [1]. Therefore, organizations need to promote innovative technologies proactively and empower their professionals to be adequately trained to successfully implement AI-based tools in their practice [1]. A concerted, deliberate approach is required to incorporate these new technologies, both effectively and compassionately, at an individual level and within the culture and operations of an organization [1].

A number of potential barriers to implementing these technologies exist; the 3 main limitations identified include regulatory, economic, and organizational culture issues [15]. Regulatory approval [16] is needed to adopt AI technologies in clinical settings, and potential liabilities in using these technologies for patient care must be considered, as well as the safety, efficacy, and transparency of AI algorithms for clinical decision-making [17,18]. Regulatory issues can also come into play when it comes to accessing data for AI adoption; multi-institution data sharing is required for algorithm improvement and validation, as well as the accompanying research ethics board and regulatory approvals [18]. To further improve adoption, these technologies will also have to be economical, supported by adequate funding [18], and seem as valuable to the organization itself. At an organizational level, the use of AI should align with the goals and strategic plans of an organization; organizations will need to assess how well the AI technology will integrate into existing systems, including data warehouses and electronic health records [18]. It may be difficult to generalize a particular AI model across different clinical contexts to a degree that would prove valuable at an organizational level while still working seamlessly and being clinically useful at the individual level [15]. Furthermore, when choosing to adopt AI technologies, organizations can either collaborate with outside vendors or create the technologies in-house, which will require the use of additional human and material resources [15].

### Objective

Deficits in AI education may be contributing to a lack of capacity in health care systems to fully integrate and adopt AI technologies to improve patient care, despite calls for AI integration as part of the National Academy of Medicine’s Quintuple Aim Model [19]. It is important to equip health care organizations and their stakeholders to have the cognitive, psychomotor, and affective skills to harness AI in enhancing and optimizing the delivery of care. This will also involve supporting AI education initiatives that are widely available for all types of HCPs. To support future AI education development, dissemination, and evaluation, it is important to assess the current situation within AI adoption in health care and further understand the extent of AI education implementation, including who is receiving AI training or education, what content is covered, how it is delivered, and whether this reflects what experts believe that AI education curricula should include. Therefore, this scoping review aims to establish a foundational understanding of education programs on AI for HCPs by determining the following:

1. What were the most effective educational approaches to enabling HCPs to harness AI in enhancing and optimizing health care delivery?
  What curricular content was delivered?
  What was the scope of content that should be delivered?
  What learning objectives were used in these approaches, using the taxonomy for learning formulated by Bloom [20]?
2. What were the enablers or barriers that contributed to the success of these programs and the implementation of AI curricula in health care education programs?
3. What outcomes were used to assess the effectiveness of the education programs, using the Kirkpatrick-Barr Framework [21]?

## Methods

### Overview

This scoping review followed the Arksey and O’Malley [22] guidelines and the PRISMA (Preferred Reporting Items for Systematic Reviews and Meta-Analyses) Extension for Scoping Reviews checklist [23,24]. The objective of this scoping review is to examine and summarize the extant literature on AI education and training for HCPs.

### Stage 1: Search Strategy

A health sciences librarian (MA) developed strategies for Ovid MEDLINE All, Ovid Embase, Ovid APA PsycINFO, Ovid Emcare Nursing, Ovid Cochrane Database of Systematic Reviews, Ovid Cochrane Central Register of Controlled Trials, EBSCO ERIC, and Clarivate Web of Science using appropriate subject headings and keywords for AI and health professions education. As a result of the widespread use of terms relating to health professions and education in health sciences literature, the decision was made to focus the searches on health professions education concepts to reduce *noise* in the results sets. Searches for these subject headings were limited to where they were the major subject heading (the most important subject heading in the database record for an item). Keywords for these concepts were only searched in the study titles, the author-assigned keywords, heading words, and journal titles, depending on the content and field availability of the database. No language or date limits were applied. The searches were run and the results were downloaded on July 7, 2020. For the complete strategies, see Multimedia Appendix 1. If the search results included conference abstracts and proceedings, a subsequent search to find any corresponding follow-up studies was conducted in Google Scholar. Finally, *pearl growing,* also known as a *hand comb* process, was conducted where all cited works in the included studies from the initial screening underwent a 2-stage screening process (title and abstract scan as well as full-text review).

### Stage 2: Study Selection

The 2-stage screening process consisted of (1) title and abstract scan and (2) full-text review. Study eligibility was determined by 2 independent reviewers, and a third reviewer was involved to resolve any conflict when consensus was not reached between the 2 reviewers. For a study to be included for full-text review and to be chosen for subsequent inclusion, the title and abstract at each stage needed to have the following attributes:

1. It discussed an actual training program or educational intervention or potential training program or educational intervention and the desired content to be covered.
2. It focused on AI.
3. It was designed or intended for HCPs (at any stage of their career).

A pilot review of 20% (595/2973) of the MEDLINE citations was conducted to establish interrater reliability. The interrater reliability threshold had a Cohen κ value of 0.70, indicating substantial agreement. Additional batches of 50 citations were reviewed until the threshold was met.

### Stage 3: Data Collection

A standardized charting form was developed to capture the following domains: article details, study details (if publication was an empirical study), education program details, and implementation factors. The subdivisions of the domains for the data extraction are outlined in Table 1.

**Table 1:** Data charting: domains and subdomains.

Domain	Subdomain
Article details	Article type, year, and country
Study details	Study design, participants, intervention, comparator, primary outcomes, and secondary outcomes
Education program details	Name, setting, participants, program delivery and curriculum, program instructors (discipline), program length, and instructor training
Implementation factors	Implementation enablers or facilitators, implementation barriers, and recommendations

### Stage 4: Synthesizing and Reporting the Results

To collate, summarize, and report on the included studies in this review, a narrative synthesis approach was used [25]. This included a numeric summary using descriptive statistics to report each domain (article details, study details, education program details, and implementation factors). For program curriculum under education program details, curriculum topics were inductively coded. Once a list of topics was generated, they were then grouped by domain using the taxonomy for learning formulated by Bloom. There are 3 domains: (1) cognitive, which refers to knowledge that learners should have, (2) psychomotor, which refers to skills learners should demonstrate and master, and (3) affective, which refers to attitudes learners should develop and incorporate into their practice [20].The study outcomes were deductively coded using the Kirkpatrick-Barr Framework [21] of educational outcomes. This framework was selected because it provided a standardized method of categorizing the type of educational outcomes reported by each study. The implementation factors subdomain was thematically analyzed by 2 independent reviewers using a priori codes. The reviewers compared coding schemes and iteratively determined overarching themes to frame their findings. For content validation, the project team members, patients, and experts in the fields of medical education and AI provided feedback on the thematic analysis.

## Results

### Search Results

**Figure 1:**
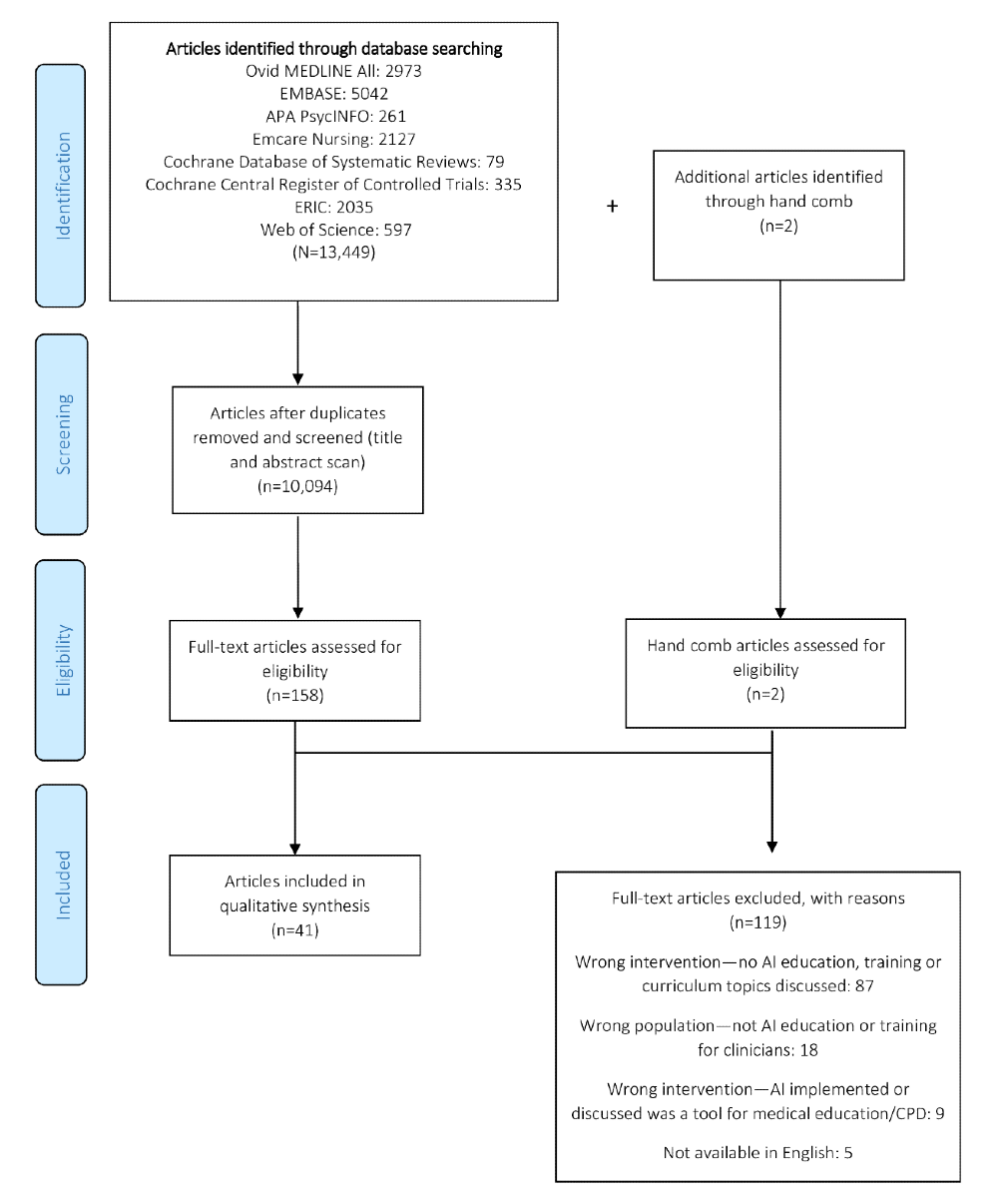
PRISMA (Preferred Reporting Items for Systematic Reviews and Meta-Analyses) flow diagram of the scoping review results. AI: artificial intelligence; CPD: continuing professional development.

The initial database search yielded 13,449 results; once duplicates were removed, the titles and abstracts of 10,094 (75.05%) unique citations were identified. From the 10,094 unique citations, we identified 41 (0.41%) articles [2,5,13,26-63], where 13 unique, existing programs [32,35,39,43,49,50,59,61-63] were mentioned in 10 (24%) articles, and the remaining 31 (76%) articles [2,5,13,26-31,33,34,36-38,40-42,44-48,51-58,60] discussed the desired or recommended curricular content. The article selection process is presented in Figure 1. Of the 10 articles that discussed an existing program, 8 (80%) were commentaries [32,43,49,50,59,61-63], 1 (10%) was a case report [39], and 1 (10%) was an empirical study [35]. Tables 2 and 3 describe the characteristics of the articles and programs included in this review.

**Table 2:** Summary of article characteristics (N=41).

Characteristics			Frequency, n (%)		References
**Study type**					
	Commentary	30 (73)		[13,26-30,32-34,36,38,41-45,47,49-53,55,56,58-63]	
	Review	6 (15)		[2,5,40,48,54,57]	
	Empirical study	3 (7)		[31,35,37]	
	Case report	1 (2)		[39]	
	Best Evidence Medical Education Guide	1 (2)		[46]	
**Publication year**					
	2013	1 (2)		[39]	
	2016	3 (7)		[35,56,61]	
	2017	2 (5)		[31,50]	
	2018	8 (20)		[5,28,32-34,42,47,53]	
	2019	16 (39)		[13,26,27,29,30,38,40,44-46,48,49,52,57,59,62]	
	2020	11 (27)		[2,36,37,41,43,51,54,55,58,60,63]	
**Country**					
	United States	23 (56)		[2,26,28-32,34,35,42,44,45,47,49-52,54,55,59,61-63]	
	Canada	4 (10)		[13,27,33,43]	
	United Kingdom	2 (5)		[37,56]	
	Other	12 (29)		[5,36,38-41,46,48,53,57,58,60]	

**Table 3:** Summary of program characteristics (N=13).

Characteristic			Frequency, n (%)		References
**Program type**					
	Workshop	2 (15)		[49,50]	
	Fellowship	3 (23)		[32,59]	
	Biomedical informatics course	2 (15)		[35,39]	
	Data science course	2 (8)		[59,61]	
	Joint course-based program	1 (8)		[59]	
	Educational summit	1 (8)		[62]	
	Certificate program	1 (8)		[43]	
	Artificial Intelligence Journal Club	1 (8)		[63]	
**Program setting**					
	Medical school	6 (46)		[39,43,59,61,62]	
	Academic hospital	4 (31)		[32,35,59]	
	National	1 (8)		[49]	
	International	2 (15)		[50,63]	
**Program length**					
	>1 year	2 (15)		[43,61]	
	>1 month	2 (15)		[39,63]	
	>1 day	1 (8)		[50]	
	≤1 day	2 (15)		[49,62]	
	Not reported	6 (46)		[32,35,59]	
**Program audience**					
	Health care professionals	12 (92)		[32,35,39,43,49,50,59,61-63]	
	Researchers or clinician scientists	2 (15)		[59,62]	
	Health care administrators	1 (8)		[62]	
	Other health disciplines	1 (8)		[63]	
**Continuum of learning^a^**					
	Undergraduate medical education	5 (39)		[39,43,59,62]	
	Postgraduate medical education	8 (62)		[32,35,49,50,62,63]	
**Program objectives^b^**					
	Cognitive or psychomotor	10 (77)		[32,35,39,43,49,50,59,61,63]	
	Affective	1 (8)		[39]	
	Both	2 (15)		[59,62]	
**Program methods**					
	Didactic	9 (69)		[32,35,39,43,49,50,59,61,62]	
	Workshop	2 (15)		[49,50]	
	Case-based	2 (15)		[49,50]	
	Discussions	2 (15)		[49,62]	
	Experiential learning	5 (39)		[43,49,59]	
	Web-based	3 (23)		[39,61,63]	
**Number of methods used**					
	1 method	5 (39)		[32,35,59,63]	
	2 methods	5 (39)		[39,43,59,61,62]	
	≥3 methods	2 (15)		[49,50]	
**Study outcomes^c^**					
	Level 1	3 (23)		[39,49,50]	
	Level 2a	3 (23)		[49,50,62]	
	Level 2b	2 (15)		[35,62]	
	None	8 (62)		[32,43,59,61,63]	

^a^There are no continuing medical education programs.

^b^Categorized based on the domains identified in the taxonomy for learning formulated by Bloom [20].

^c^Categorized based on the education outcomes identified in the Kirkpatrick-Barr Framework [21].

### What Was the Mode of Delivery?

Summaries of the individual programs can be found in Table 4. Of the 13 programs, 8 (62%) originated from the United States [32,35,49,50,59,61-63], 1 (8%) from Canada [43], 1 (8%) from France [59], and 1 (8%) from Mexico [39]. The typology described by Strosahl [64] was used to classify the educational method. Of the 13 programs, 9 (69%) had a didactic approach [32,35,39,43,50,59,61,62] in combination with discussions [62] (1/13, 8%), web-based [39,61] (2/13, 15%), workshop and case-based [50] (1/13, 8%), and experiential learning [43] (1/13, 8%). Of the 13 programs, 10 (77%) were taught in an academic setting [32,35,39,43,59,61,62].

**Table 4:** Summary of program details.

Program name or first author; country; host institution; specialty; program length	Program setting					Curriculum delivery methods						
	Medical school	Academic hospital	National	International	Didactic		Workshop	Case-based	Discussion	Experiential learning	Web-based	
Artificial Intelligence Journal Club; United States; American College of Radiology; Radiology; monthly for 1 hour [63]				✓								✓
Educational Summit; United States; Duke University Medical Center; NS^a^; <1 day [62]	✓				✓				✓			
Health Care by Numbers; United States; New York University; NS; 3 years [61]	✓				✓							✓
Joint course-based program; France; Gustave Roussy with École des Ponts ParisTech and CentraleSupélec; NS; NR^b^ [59]	✓				✓							
Fellowship; United States; Emory University School of Medicine; Radiology; NR [59]		✓								✓		
Fellowship; United States; Hospital of the University of Pennsylvania; Imaging Informatics; NR [59]		✓								✓		
Elective courses; United States; Carle Illinois College of Medicine; NS; NR [59]	✓				✓					✓		
Introduction to Comparative Effectiveness Research and Big Data Analytics for Radiology; United States; New York University School of Medicine; medical imaging; 2 days [50]				✓	✓		✓	✓				
Kinnear; United States; University of Cincinnati; NS; <1 day [49]			✓				✓	✓	✓	✓		
Computing for Medicine certificate program; Canada; University of Toronto, Faculty of Medicine; NS; 2 years [43]	✓				✓					✓		
The National Autonomous University of Mexico, Faculty of Medicine, biomedical informatics education; Mexico; University of Mexico’s Faculty of Medicine; NS; 2 one-semester courses [39]	✓				✓							✓
Formalized bioinformatics education; United States; Baylor Scott and White Medical Center; medical imaging; NR [35]		✓			✓							
National Cancer Institute–Food and Drug Administration Information Exchange and Data Transformation fellowship in oncology data science; United States; National Cancer Institute; medical imaging; NR [32]		✓			✓							

^a^NS: specialty not specified.

^b^NR: not reported.

### Target Audience

**Figure 2:**
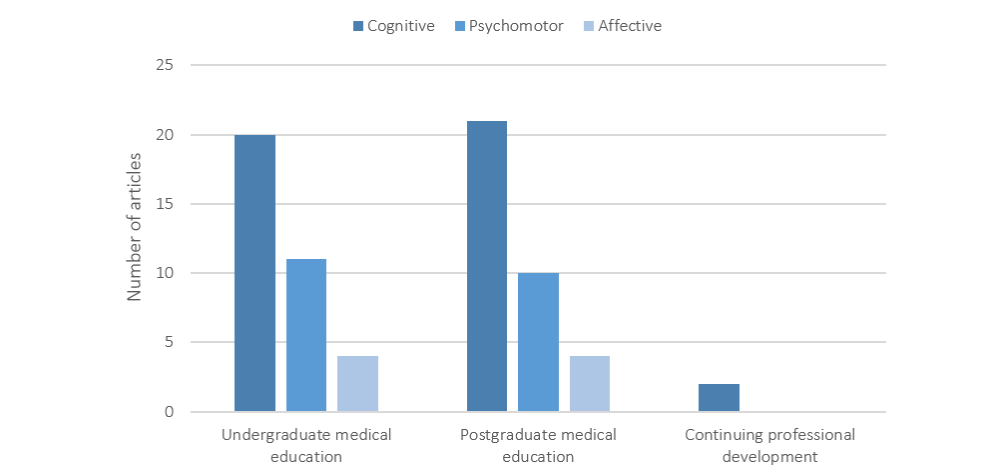
Number of articles in each curriculum topic domain grouped by target audience.

There were 3 types of HCPs identified in the 41 reviewed papers: physicians [41,43,46,52,59,63] (6/41, 15%), nurses [31,52] (2/41, 5%), and radiology technologists [5] (1/41, 2%). In addition, 2 specific specialties were identified: medical imaging [5,26,32-34,37,42,48,50,51,55,58,63] (13/41, 32%) and cardiology [56,61] (2/41, 5%), with others not being specified [2,13,27-31,35,36,38-41,43-47,49,52-54,57,59,60,62] (26/41, 63%). Figure 2 illustrates the type of curriculum topics covered in the continuum of learning for clinicians, which includes undergraduate medical education [2,13,28-30,33,35-37,39-41,43,44,47,53,57,61,62] (20/41, 49%), postgraduate medical education [5,26,32-35,41,42,48-51, 54-58,62,63] (19/41, 46%), and continuing professional development [5,57] (2/41, 5%). Other nonclinical professionals include researchers [5,27,33,59,62] (5/41, 12%), health care administrators [27,33,45,52,62] (5/41, 12%), and computer and data scientists [27,33,52,63] (4/41, 10%).

### What Content Was Covered?

From these papers, the program curriculum and desired or recommended content mentioned included topics on using AI, interpreting AI, and explaining results from AI, as framed by McCoy et al [43]. A description of each curricular topic can be found in Table 5.

Of these 16 curricular topics, 9 (56%) fell under cognitive domain, 6 (38%) under psychomotor domain, and 1 (6%) under affective domain, and most of them were mentioned both by papers that discussed current education programs and commentaries that discussed what HCPs should be learning. The curricular topics were categorized into the 3 domains identified in the taxonomy for learning formulated by Bloom [20]. Table 6 displays the curricular topics that were unique to 24% (10/41) of the papers [32,35,39,43,49,50,59,61-63] that described what AI programs currently teach, 76% (31/41) of the papers [2,5,13,26-31,33,34,36-38,40-42,44-48,51-58,60] that described what AI programs should teach as part of their curriculum, and those that outlined both what was taught and what should be taught.

**Table 5:** Curriculum focus and objectives.

Themes (framed by McCoy et al [43]) and topic		Description	Number of studies	References
**Using AI^a^**				
	Fundamentals of AI	An overview of all stages of model development, translation, and use in clinical practice. Specifically, this would cover nomenclature and principles such as data collection and transformation, algorithm selection, model development, training and validation, and interpreting model output	20	[5,26,27,32,34,36,37,39-41,43,46,47,51,52,55,57-59,62]
	Fundamentals of health care data science	Fundamental understanding of the environment supported by AI. This includes an overview of biostatistics, big data, data streams available, and how algorithms and machine learning use and process data	20	[5,13,26-36,41,42,45,49,51,52,54]
	Fundamentals of biomedical informatics	An overview of essential concepts such as nomenclature (information and knowledge taxonomy), structure and function of computers, information and communications technology, standards in biomedical informatics, and technology evaluation	1	[39]
	Multidisciplinary collaboration	Learning how to partner and communicate with experts in engineering and data science to ensure clinical relevance and accuracy of AI systems	13	[26,29,31,33,43,45,51-54,57,58,62]
	Applications of AI	Providing examples of AI that have been implemented in health care settings to understand the impact of technologies that incorporate AI	11	[2,32,39,40,44-46,51,52,55,57]
	Implementation of AI in health care settings	Understanding how to embed AI tools into clinical settings and workflows. Specifically, this includes requirements for clinical translation and interpretation of model outputs	9	[27,30,32-34,41,45,57,62]
	Strengths and limitations of AI	Understanding the value, pitfalls, weaknesses and potential errors or unintended consequences that may occur when using AI tools	13	[26,30,32-34,37,41,45,51,52,55,58,62]
	Ethical considerations	Understanding and building awareness of ethics, equity, inclusion, patient rights, and confidentiality when using AI tools	13	[5,26,28-30,33,36,39,41,42,46,54,58]
	Legal considerations and governance strategy	Understanding data governance principles, regulatory frameworks, legislation, policy on using data and AI tools, as well as liability or intellectual property issues	7	[27,30,39,41,45,51,58]
	Economic considerations	“Understanding of how business or clinical processes will be altered through the integration of AI technologies into health care” [58] as well as commercialization	2	[26,33]
**Interpreting results from** **AI**				
	Medical decision-making	Understanding decision science and probabilities from AI diagnostic and therapeutic algorithms to then meaningfully apply them in clinical decision-making	8	[13,26,28-31,39,51]
	Data visualization	Understanding how to present and describe outputs from AI tools	4	[27,30,52,54]
	Product development projects	Hands on experience to develop, test, and validate AI algorithms with real medical data	2	[52,54]
**Explaining results from** **AI**				
	Communicating with patients	Mastering how to communicate results with patients in a personalized and meaningful way and discuss the use of AI in the medical decision-making process	8	[5,28-30,32,36,43,46]
	Compassion and empathy	Cultivating and expressing empathy and compassion when communicating with patients	4	[28-30,36]
	Critical appraisal	Understanding how to evaluate AI diagnostic and therapeutic algorithms	7	[2,34,40,43,51,54,59]

^a^AI: artificial intelligence.

**Table 6:** Illustration of the cognitive, psychomotor, and affective domains between what programs currently teach as part of the artificial intelligence (AI) curriculum and what programs should teach.

Competencies	What programs currently teach	Similarities between the current program and recommended program topics	What programs should teach
Cognitive	Informatics	Fundamentals of AI Implementation of AI in health care settings Big data Data science Machine learning Statistics Multidisciplinary collaboration Strengths and limitations of AI	Challenges with AI AI applications EHR^a^ fundamentals Predictive analytics Ethics and legal Issues Data governance Economic considerations
Psychomotor	Leadership	Analytical Problem solving Interpretation Communication Critical appraisal Medical decision-making	Cultivation of compassion and empathy Product development Data visualization
Affective	Perception of humanistic AI-enabled care	Beliefs about how AI will affect future of health careers and patient care	Change management Adoption of AI Create and sustain a culture of trust and transparency with stakeholders and patients

^a^EHR: electronic health record.

### Cognitive Domain

Of the 41 papers, 20 (49%) [5,26,32,34,36,37,39-41,43, 45-47,50,52,55,57-59,62] highlighted the importance of providing HCPs with a baseline understanding of AI and 10 (24%) [32,35,39,43,49,50,59,61-63] recommended teaching them AI applications. The studies focused on various applications of AI, including diagnostic systems, data gathering, assessment and use, clinical applications, and personalized care. In addition, many of the papers reported that medical curricula should integrate fundamentals of health care data science [5,13,26-36,42,45,49,50,52,54] (19/41, 46%), including, but not limited to, big data and bioinformatics. Matheny et al [45] stated that data science curricula should encompass how to form multidisciplinary development teams to improve the value of AI and to be aware of the ethics, equity, diversity, and inclusion principles at play and the inadvertent ramifications that may result from AI implementation. The studies also focused on statistics, ML with model development, model translation and use in clinical knowledge, data extraction, and applications for visualization of patients. Familiarity with ML vocabulary and a basic understanding of the methodology (algorithms and machine gathering and process of data) were deemed important to understand this rapidly emerging field.

### Psychomotor Domain

Most of the papers focused on clinicians being able to effectively analyze the data [2,5,31,34,35,40,43,46,50,54,55,58,59,61,63] (15/41, 37%) to identify trends and efficiency correlations. As highlighted by Balthazar et al [63] and Forney and McBride [55], it is imperative to learn how to evaluate the efficacy and precision of AI applications. This point was reinforced in a review conducted by Park et al [40] that stated medical students should be able to validate the clinical accuracy of algorithms. HCPs will need to become accustomed and understand how to embrace real-time health information to help make decisions in their practice setting [61]. Of the 41 papers, 8 (20%) discussed the significance of understanding and interpreting the findings with a reasonable degree of accuracy, including awareness of source error, bias, or clinical irrelevance [13,28-31,39,47,50]. Moreover, the study findings described problem-solving [35,38,60] (3/41, 7%) as a critical skill, entailing the management and application of several distinct resources. Clinicians will need to become adept in communicating the results and processes [5,28-30,32,36,43,46] (8/41, 20%) with patients in a personalized and meaningful manner. Cultivating and expressing empathy and compassion [28-30,36] (4/41, 10%) when communicating with the patient was emphasized in several studies.

### Affective Domain

Of the 41 papers, 8 (20%) stressed that HCPs should have the attitude to harness AI tools effectively to improve outcomes for patients and their communities [27,30,37,52,55,58,59,62]. Wiljer and Hakim [27] asserted the importance of breaking the mass stereotypes about AI as an initial step. It is essential that professionals perceive AI as augmenting their delivery of care, rather than taking over different aspects of the health care system [62]. Forney and McBride [55] stated that clinicians are not as likely to perceive AI as a threat if they are able to see the wide array of AI tools and the impact these tools have on workflow and patient care. Furthermore, Sit et al [37] mentioned that medical students are not as likely to be discouraged from pursuing certain specialties when they are presented with use cases and understand the boundaries of AI tools; almost half of the respondents believed the misconception that because of AI, certain specialists such as radiologists will become obsolete in the near future. Moreover, Brouillette [59] mentioned the need for collaborative programs among medical students, computer science students, and engineering students, where they can better understand each other’s disciplines. A few papers recommended that future AI programs should integrate change management and establish a culture of trust and transparency with relevant stakeholders, which will support organizations to more rapidly adopt and implement AI technologies within the health care ecosystem [27,30]. Thus, it is vital to help organizations manage change at a rate in pace with the rapid advancement of technology.

### What Were the Critical Implementation Factors?

#### Enablers

The factors identified as contributing, or potentially contributing, to the success and implementation of these programs include promoting interfaculty collaboration [39,54,57] and working within existing regulatory structures [28,37,39,57]. Not all institutions have clinical faculty who also have experience with data science; hence, there is a need in both practice and teaching for collaboration with data science faculty. Promoting interfaculty collaboration was described in the studies as the sharing of expertise among faculty members, thus creating a multidisciplinary team [39,54,57]. Collaborative teaching by clinical and nonclinical instructors may increase the educational value when preparing future HCPs and also provide data science support to faculty [39,54]. Another facilitator to implementation is working within existing regulatory structures. Curriculum changes require the support of existing accreditation and regulatory bodies [28]. A few papers discussed the need for the integration of mandatory AI coursework and assessments with the current curricula [39,57]. Hence, this could address varying AI literacy levels; enhancing knowledge of AI will increase the likelihood that it will be used in practice settings [37].

#### Barriers

Overall, 2 major barriers were identified that could potentially impede an organization’s implementation efforts: (1) varying levels of AI literacy among faculty in designing curricula [54,57] and (2) lack of infrastructure to integrate AI into the current curriculum [34,39,50,54]. Varying levels of AI literacy among faculty and curriculum leaders was discussed as a major barrier that encumbers the implementation of AI programs. Of the 41 papers, 2 (5%) discussed how faculty have limited knowledge of AI fundamentals (eg, big data or data science) and software, as well as limited time to teach [54,57]. There is a lack of technical expertise to design AI-based curricula [49,57]. Moreover, a few studies voiced concerns about the lack of infrastructure to integrate AI into the curriculum. Some studies highlighted that the existing curricula are comprehensive and complex and additional content on AI will increase the course load [34,50,54]. Academic institutions are faced with several encumbrances such as faculty retirement, staff not being well-versed in AI, and inadequate financial resources [54]. Finally, integrating the AI content into existing curricula can be an impediment for many organizations [39].

### What Measures and Outcomes Were Used to Assess the Effectiveness of Education Programs?

Of the 41 papers, 5 (12%) presented the results of their training evaluation [35,39,49,50,62]. As the educational approaches varied across studies, each approach will be briefly discussed (Table 7), followed by the measures and outcomes associated with each educational initiative. Categorized according to the Kirkpatrick-Barr Framework, the outcomes were either level 1 (ie, learner reaction and satisfaction with the education) [39,49,50], level 2a (ie, change in attitude) [49,50,62], or level 2b (ie, change in knowledge or skill) [35,62]. There were no outcomes that could be categorized as level 3 or level 4; thus, the program evaluations did not comment on the change in behavior or affect at the organizational level or on patient outcomes.

**Table 7:** Summary of the 5 papers that assessed the effectiveness of the education program.

Programs and authors		Measure	Actual outcomes
**Educational summit**			
	Barbour et al 2019, [62]	Conducted a 5-question before-and-after poll of those attending our educational summit	Level 2a: Baseline beliefs about how AI^a^ will affect the future of health care careers and patient care were similarly positive before and after the event Level 2a: At arrival, 70% of the attendees felt that AI would make health care less humanistic; 50% left the summit feeling neutral Level 2a: We did not observe a meaningful shift in attitudes regarding the desire to take a leadership role in developing or implementing AI Level 2b: At arrival, 40% of the attendees believed that they had a poor baseline understanding of AI’s role in health care; 90% left the summit with an enhanced understanding of the topic
**Workshops**			
	Kang et al 2017, [50]	A survey was designed to capture residents’ opinions after their minicourse, covering 5 major areas of interest: How helpful the minicourse was as an introduction to CER^b^ and big data research (on a 5-point scale, with 5 indicating very helpful) Whether the residents would likely pursue further educational or research opportunities in CER Whether the residents had prior educational or research exposure to CER Whether a mentor was available for CER at their home institutions The importance of CER and big data research to the field of radiology (on a 5-point scale, with 5 indicating very important)	Level 1: 90% of the residents reported that the course was helpful or very helpful Level 1: 94% of the participants felt that the lectures were of high or very high quality Level 2a: 82% reported that they planned to pursue additional educational or research training in CER or big data analytics after the course Level 2a: 98% of the respondents felt that health services and big data research are important or very important for the future of radiology
	Kinnear et al 2019, [49]	Evaluations were conducted on a 5-point Likert scale	Level 1: The average weighted rating on a 5-point Likert scale over the 3 years for the prompt “Overall satisfaction with the session” was 4.32 out of 5 Level 2a: The participants reported an increase in confidence to use this knowledge to teach residents in the coming academic year
**Biomedical informatics course within medical education**			
	Sanchez-Mendiola et al 2013, [39]	Administered a program-evaluation anonymous survey to the students at the end of the course, a 41-item questionnaire that explored several aspects of the program	Level 1: Overall opinion of the students regarding the different elements of the program was good to excellent for educational activities, course resources, and perception of clinical relevance
	Sybenga et al 2016, [35]	Competency of senior residents on the basis of their project results was evaluated by staff during a multidisciplinary conference	Level 2b: After introductory education in big data analysis concepts, the residents were able to rapidly analyze large sets of data to answer simple questions Level 2b: The senior residents were able to engage in complex problem solving requiring management and application of multiple seemingly unrelated resources and successfully present these results

^a^AI: artificial intelligence.

^b^CER: comparative effectiveness research.

## Discussion

### Current State of AI Education Programs

This review identified pivotal knowledge gaps in our understanding of effective AI education programs for HCPs. The gaps identified through this review illustrated the limited AI education and training opportunities available for HCPs and thus emphasized the necessity of curating further AI education programs targeted to HCPs. The existing programs tend to focus only on the development and implementation of AI; yet, it is essential to also prepare HCPs to not only work with AI but also to advance AI for health and clinical decision-making. AI education programs should be designed in a way that enables HCPs to not only safely adopt these technologies, but also to adapt and shift their scope of practice to stay relevant. A significant and meaningful change to AI curricula in health care will only occur by increasing AI literacy among HCPs and by providing them with the ability to leverage relevant digital and data-driven decision-making tools. Although the studies demonstrate that efforts are being made to evaluate the outcomes of AI-related education initiatives, there is a lack of consistency in the measures for a comprehensive assessment of these outcomes. Most of the papers used self-constructed and nonvalidated instruments and delineated their findings in qualitative terms. Given the variety of instruments that have been employed in the studies, the absence of a standard, comprehensive tool impedes the integration and synthesis of findings across the studies. The guiding principles provided in this review will also hopefully inform future development and design of these programs.

### Critical Implementation Factors

A lack of infrastructure to integrate AI content into current curricula could hinder the development of these types of programs; some of the programs described embedded their content within existing professional certifying bodies’ infrastructure to facilitate content development. The Royal College of Physicians and Surgeons of Canada, in particular, further emphasizes the need for these regulatory strategies, which are currently in process but not yet in practice [65,66]. The promotion of multidisciplinary collaboration was indicated as an enabler of content delivery; yet, varying levels of AI literacy among faculty could impede successful delivery of AI content [54,57]. Curricular adaptations and building an infrastructure for AI technologies could be helpful to HCPs wanting to adopt AI to improve patient care; this includes improvements in the types of health care data available for AI education [67]. Of note, much of the health data generated are often inaccessible to researchers and limited by regulatory or infrastructure-level barriers, including institutional ethics approvals and data-sharing agreements [67]. The use of deidentified data, security, and privacy controls could potentially widen the scope of access; broader collaboration with multidisciplinary experts could also help to establish secure data networks to improve use and access of health care data [67]. Lower levels of AI literacy could be augmented by standardizing competency statements and engaging and training faculty in e-learning, for instance [68-70]. The World Health Organization’s Global Strategy on Digital Health further suggests that the barriers to AI adoption need to be addressed at the systems level and all aspects of implementation should be considered.

Our recommendations have been formed into guiding principles that could be used to guide the development of future AI curricula or to incorporate AI education into existing curricula.

### Guiding Principles

#### Principle 1: Need for Regulatory Strategies

Many studies discussed that working within the existing regulatory structure can hinder the implementation of AI education initiatives. Faculty can be inhibitors to changing curricula that were initially developed to prepare students for their national board examinations [28,30]. In addition, teaching approaches may be too outdated to incorporate new and emerging technologies [29] into the changing digital and AI landscape. New regulatory strategies will be required, and organizations will have to prioritize developing a workforce that not only has the knowledge and skills to provide care with these tools, but also the competencies to rapidly learn and adapt. The studies also highlighted that accrediting bodies can be a roadblock to change [27-29]. Wartman and Combs [28] stated that to prepare future care providers for AI-enabled care, there is a need for accreditors to move beyond traditional models (based on fact memorization and clinical clerkships) and be willing to innovate and consider new approaches to lifelong learning.

#### Principle 2: Multidisciplinary Approach to Design and Delivery

The rapidly evolving nature of the field and the dynamic regulatory, legal, and economic landscape may hinder the implementation of an AI curriculum and thus affect the deployment of AI tools in clinical practice. An initial AI curriculum must be developed iteratively because many of these areas still entail considerable research and advancement [26], ensuring that new knowledge gains and policy changes are reflected within the curriculum. This finding was reinforced in a paper by Wiljer and Hakim [27]. The authors reported that AI applications have not yet developed to a level of complexity and clinical value because many of these applications are currently in the research and development stages.

Wiens et al [71] stated that successful ML deployment entails assembling experts and stakeholders from various disciplines, including knowledge experts, decision-makers, and users. The approach to curriculum redesign will need to focus on multiple disciplines and levels of training; curricula should be specialized to the needs of various individuals such as health care researchers, clinicians, and quality improvement teams [44]. Therefore, the development of an AI-based curriculum should involve a multidisciplinary team comprising health system leaders, frontline providers, data scientists, patients, and education experts to ensure accuracy and clinical relevance of the curriculum [57,71]. It is imperative for all stakeholders and experts in the field to work collaboratively to understand and address the potential biases, thus reducing the existing social inequalities and ultimately leading to optimal care for all patients [71].

#### Principle 3: Competence-Based Curriculum Design

To influence the development of their future practice, it is essential for HCPs to have a foundational level of AI competencies and skills [27]. Education should be designed in a manner that teaches HCPs to work with, and understand, the AI they use in their clinical practice. Furthermore, a level of baseline competencies in AI should allow trainees to make significant contributions to health policy decisions related to their scope of practice [50]. AI will likely contribute significantly to the medical practice of the future; therefore, fundamentals and applications of AI tools and terminologies should be integrated into medical school curricula. Specifically, training current and future physicians on how to use these tools to provide quality health care, while taking into account the limitations and ethical implications of such technologies, will be useful [43]. In addition to medical learners and physicians, medical teachers need to be trained to deliver this innovative AI curriculum content; this is a shift that needs to occur without delay, given the steep learning curve ahead [36]. Paranjape et al [41] recommended a staged approach to educating future care providers about AI and its application in health care that spans from undergraduate to continuing medical education.

**Figure 3:**
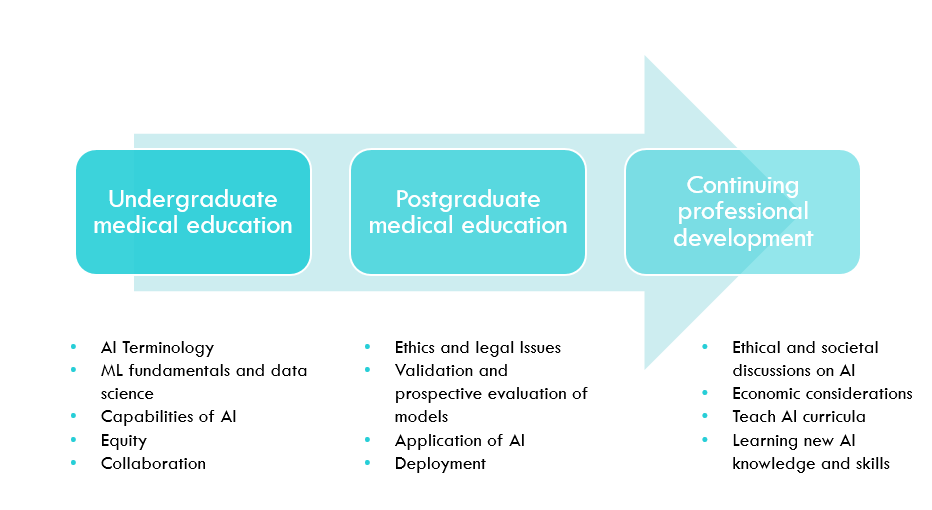
Ideal flow of key concepts for AI education curricula. The terms have been defined in the Results section. AI: artificial intelligence; ML: machine learning.

On the basis of the findings of this review, an ideal flow of AI concepts could be split across the 3 stages of medical education defined by Oxford Medicine: undergraduate medical education, postgraduate medical education, and continuing professional development (Figure 3) [72]. Undergraduate medical education should be focused on HCPs becoming familiar with AI terminology, the fundamentals of ML and data science, capabilities of AI, and how to identify opportunities and applications in health where AI would be appropriate with a health equity lens. During postgraduate medical education, emphasis should be placed on how to engage in validation and prospective evaluation of models, as well as deployment. Ethical and legal considerations, including governance strategy development, should be explored in more depth. Finally, during continuing professional development, providers should be involved in facilitating ethical and societal discussions, teaching AI courses, and keeping abreast of new AI knowledge and skills as well as teaching methods.

#### Principle 4: Patient-Clinician Interaction

In the age of AI-enabled care, HCPs must consider the potential impact of the patient and clinician interaction as well as the strategies for improving the quality of care delivered in a technology-enabled environment [13,27]. Li et al [13] stated that health professions education should teach and cultivate altruism and compassion, unique skills to humans that are integral to the emergence of AI applications. This will ensure that HCPs are not disrupted by novel tools. To equip themselves to use AI in practice, care providers should develop competencies that allow them to differentiate between credible and false information in their delivery of care [40]. Similar to the situation in other industries, the challenge of adopting and implementing AI in health care will lead to winners and laggards [48]. In the successful adoption of AI, HCPs should engage with their patients because these interactions will be important to complement the technical expertise of AI as AI transforms the health care milieu [48].

### Limitations

Our scoping review findings should be examined in the context of the following limitations. Because of the nature of the scoping review, the quality of each identified study was not assessed. Given the nature of the topic being investigated, we excluded studies that discussed AI as a tool for medical education or continuing professional development. Only studies in English were included. In addition, the educational approaches varied across the studies; thus, we were unable to conduct formal comparisons among the curricula to determine which were effective. However, reviewing the literature enabled us to identify the gaps in current education programs and provide insights and best practices to guide future education efforts. As this review was inclusive of all types of studies and focused on a breadth of literature, the depth in reporting of education program details was inconsistent and varied based on the scope of the study.

### Conclusions

With the inevitable progression of health care digitization, health professions education should foster unique human abilities, which will complement these emerging technologies. This review provided an overview of the current state of AI in health professions education and future directions on preparing care providers for the era of AI in health care. Future education efforts should focus on the development of regulatory strategies, a multidisciplinary approach to curriculum redesign, a competency-based curriculum, and patient-clinician interaction.

## Appendix

Multimedia Appendix 1 Database search strategies.

## Abbreviations

AI: artificial intelligence
HCP: health care professional
ML: machine learning
PRISMA: Preferred Reporting Items for Systematic Reviews and Meta-Analyses

## References

[1] Brinker S Martec's Law: technology changes exponentially, organizations change logarithmically 2013. Chief Martec.

[2] Sapci AH, Sapci HA (2020). Artificial intelligence education and tools for medical and health informatics students: systematic review. JMIR Med Educ.

[3] Topol EJ (2019). High-performance medicine: the convergence of human and artificial intelligence. Nat Med.

[4] Berner ES, McGowan JJ (2010). Use of diagnostic decision support systems in medical education. Methods Inf Med.

[5] SFR-IA Group, CERF, French Radiology Community (2018). Artificial intelligence and medical imaging 2018: French Radiology Community white paper. Diagn Interv Imaging.

[6] Mattessich S, Tassavor M, Swetter SM, Grant-Kels JM (2018). How I learned to stop worrying and love machine learning. Clin Dermatol.

[7] Meek RD, Lungren MP, Gichoya JW (2019). Machine learning for the interventional radiologist. AJR Am J Roentgenol.

[8] The impact of digital health technologies on the future of medical specialties in one infographic. The Medical Futurist.

[9] (2018). How artificial intelligence is transforming the world. Brookings.

[10] Fridsma DB (2018). Health informatics: a required skill for 21st century clinicians. BMJ.

[11] Collado-Mesa F, Alvarez E, Arheart K (2018). The role of artificial intelligence in diagnostic radiology: a survey at a single radiology residency training program. J Am Coll Radiol.

[12] Moore JH, Boland MR, Camara PG, Chervitz H, Gonzalez G, Himes BE, Kim D, Mowery DL, Ritchie MD, Shen L, Urbanowicz RJ, Holmes JH (2019). Preparing next-generation scientists for biomedical big data: artificial intelligence approaches. Per Med.

[13] Li D, Kulasegaram K, Hodges BD (2019). Why we needn't fear the machines: opportunities for medicine in a machine learning world. Acad Med.

[14] Han E, Yeo S, Kim M, Lee Y, Park K, Roh H (2019). Medical education trends for future physicians in the era of advanced technology and artificial intelligence: an integrative review. BMC Med Educ.

[15] Singh RP, Hom GL, Abramoff MD, Campbell JP, Chiang MF, AAO Task Force on Artificial Intelligence (2020). Current challenges and barriers to real-world artificial intelligence adoption for the healthcare system, provider, and the patient. Transl Vis Sci Technol.

[16] Varghese J (2020). Artificial intelligence in medicine: chances and challenges for wide clinical adoption. Visc Med.

[17] Holm EA (2019). In defense of the black box. Science.

[18] He J, Baxter SL, Xu J, Xu J, Zhou X, Zhang K (2019). The practical implementation of artificial intelligence technologies in medicine. Nat Med.

[19] Cox M, Blouin AS, Cuff P, Paniagua M, Phillips S, Vlasses PH (2017). The role of accreditation in achieving the quadruple aim. National Academy of Medicine.

[20] Hoque M (2017). Three domains of learning: cognitive, affective and psychomotor. Academic Research - What the research project is intended to achieve.

[21] Shen N, Yufe S, Saadatfard O, Sockalingam S, Wiljer D (2017). Rebooting Kirkpatrick: integrating information system theory into the evaluation of web-based continuing professional development interventions for interprofessional education. J Contin Educ Health Prof.

[22] Arksey H, O'Malley L (2005). Scoping studies: towards a methodological framework. Int J Soc Res Methodol.

[23] Tricco AC, Lillie E, Zarin W, O'Brien KK, Colquhoun H, Levac D, Moher D, Peters MD, Horsley T, Weeks L, Hempel S, Akl EA, Chang C, McGowan J, Stewart L, Hartling L, Aldcroft A, Wilson MG, Garritty C, Lewin S, Godfrey CM, Macdonald MT, Langlois EV, Soares-Weiser K, Moriarty J, Clifford T, Tunçalp Ö, Straus SE (2018). PRISMA Extension for Scoping Reviews (PRISMA-ScR): checklist and explanation. Ann Intern Med.

[24] PRISMA for scoping reviews. PRISMA.

[25] Colquhoun HL, Levac D, O'Brien KK, Straus S, Tricco AC, Perrier L, Kastner M, Moher D (2014). Scoping reviews: time for clarity in definition, methods, and reporting. J Clin Epidemiol.

[26] Wood MJ, Tenenholtz NA, Geis JR, Michalski MH, Andriole KP (2019). The need for a machine learning curriculum for radiologists. J Am Coll Radiol.

[27] Wiljer D, Hakim Z (2019). Developing an artificial intelligence-enabled health care practice: rewiring health care professions for better care. J Med Imaging Radiat Sci.

[28] Wartman SA, Combs CD (2018). Medical education must move from the information age to the age of artificial intelligence. Acad Med.

[29] Wartman S, Combs C (2019). Reimagining medical education in the age of AI. AMA J Ethics.

[30] Wartman SA (2019). The empirical challenge of 21st-century medical education. Acad Med.

[31] Topaz M, Pruinelli L (2017). Big data and nursing: implications for the future. Stud Health Technol Inform.

[32] Thompson RF, Valdes G, Fuller CD, Carpenter CM, Morin O, Aneja S, Lindsay WD, Aerts HJ, Agrimson B, Deville C, Rosenthal SA, Yu JB, Thomas CR (2018). Artificial intelligence in radiation oncology: a specialty-wide disruptive transformation?. Radiother Oncol.

[33] Tang A, Tam R, Cadrin-Chênevert A, Guest W, Chong J, Barfett J, Chepelev L, Cairns R, Mitchell JR, Cicero MD, Poudrette MG, Jaremko JL, Reinhold C, Gallix B, Gray B, Geis R, Canadian Association of Radiologists (CAR) Artificial Intelligence Working Group (2018). Canadian association of radiologists white paper on artificial intelligence in radiology. Can Assoc Radiol J.

[34] Tajmir SH, Alkasab TK (2018). Toward augmented radiologists: changes in radiology education in the era of machine learning and artificial intelligence. Acad Radiol.

[35] Sybenga A, Zreik RT, Mohammad A, Rao A Big Data: bioinformatics education during residency demonstrates immediate value. Nature Publishing Group.

[36] Srivastava TK, Waghmare L (2020). Implications of Artificial Intelligence (AI) on dynamics of medical education and care: a perspective. J Clin Diagnos Res.

[37] Sit C, Srinivasan R, Amlani A, Muthuswamy K, Azam A, Monzon L, Poon DS (2020). Attitudes and perceptions of UK medical students towards artificial intelligence and radiology: a multicentre survey. Insights Imaging.

[38] Saqr M, Tedre M (2019). Should we teach computational thinking and big data principles to medical students?. Int J Health Sci (Qassim).

[39] Sánchez-Mendiola M, Martínez-Franco AI, Lobato-Valverde M, Fernández-Saldívar F, Vives-Varela T, Martínez-González A (2015). Evaluation of a Biomedical Informatics course for medical students: a pre-posttest study at UNAM Faculty of Medicine in Mexico. BMC Med Educ.

[40] Park SH, Do K, Kim S, Park JH, Lim Y (2019). What should medical students know about artificial intelligence in medicine?. J Educ Eval Health Prof.

[41] Paranjape K, Schinkel M, Nannan Panday R, Car J, Nanayakkara P (2019). Introducing artificial intelligence training in medical education. JMIR Med Educ.

[42] Nguyen GK, Shetty AS (2018). Artificial intelligence and machine learning: opportunities for radiologists in training. J Am Coll Radiol.

[43] McCoy LG, Nagaraj S, Morgado F, Harish V, Das S, Celi LA (2020). What do medical students actually need to know about artificial intelligence?. NPJ Digit Med.

[44] Mathur P, Burns M (2019). Artificial intelligence in critical care. Int Anesthesiol Clin.

[45] Matheny ME, Whicher D, Thadaney Israni S (2020). Artificial intelligence in health care: a report from the national academy of medicine. JAMA.

[46] Masters K (2019). Artificial intelligence in medical education. Med Teach.

[47] Kolachalama VB, Garg PS (2018). Machine learning and medical education. NPJ Digit Med.

[48] Kobayashi Y, Ishibashi M, Kobayashi H (2019). How will "democratization of artificial intelligence" change the future of radiologists?. Jpn J Radiol.

[49] Kinnear B, Hagedorn P, Kelleher M, Ohlinger C, Tolentino J (2019). Integrating Bayesian reasoning into medical education using smartphone apps. Diagnosis (Berl).

[50] Kang SK, Lee CI, Pandharipande PV, Sanelli PC, Recht MP (2017). Residents' introduction to comparative effectiveness research and big data analytics. J Am Coll Radiol.

[51] Kang J, Thompson RF, Aneja S, Lehman C, Trister A, Zou J, Obcemea C, El Naqa I (2021). National cancer institute workshop on artificial intelligence in radiation oncology: training the next generation. Pract Radiat Oncol.

[52] Jeffery A (2019). ANI emerging leader project: identifying challenges and opportunities in nursing data science. Comput Inform Nurs.

[53] Gorman D, Kashner TM (2018). Medical graduates, truthful and useful analytics with big data, and the art of persuasion. Acad Med.

[54] Foster M, Tasnim Z (2020). Data science and graduate nursing education: a critical literature review. Clin Nurse Spec.

[55] Forney MC, McBride AF (2020). Artificial intelligence in radiology residency training. Semin Musculoskelet Radiol.

[56] Evans J, Banerjee A (2016). Global health and data science: future needs for tomorrow’s cardiologist. Br J Cardiol.

[57] Chan KS, Zary N (2019). Applications and challenges of implementing artificial intelligence in medical education: integrative review. JMIR Med Educ.

[58] Chamunyonga C, Edwards C, Caldwell P, Rutledge P, Burbery J (2020). The impact of artificial intelligence and machine learning in radiation therapy: considerations for future curriculum enhancement. J Med Imaging Radiat Sci.

[59] Brouillette M (2019). AI added to the curriculum for doctors-to-be. Nat Med.

[60] Briganti G, Le Moine O (2020). Artificial intelligence in medicine: today and tomorrow. Front Med (Lausanne).

[61] Bhavnani SP, Muñoz D, Bagai A (2016). Data science in healthcare: implications for early career investigators. Circ Cardiovasc Qual Outcomes.

[62] Barbour AB, Frush JM, Gatta LA, McManigle WC, Keah NM, Bejarano-Pineda L, Guerrero EM (2020). Artificial intelligence in health care: insights from an educational forum. J Med Educ Curric Dev.

[63] Balthazar P, Tajmir SH, Ortiz DA, Herse CC, Shea LA, Seals KF, Cohen-Addad D, Purkayastha S, Gichoya JW (2020). The Artificial Intelligence Journal Club (#RADAIJC): a multi-institutional resident-driven web-based educational initiative. Acad Radiol.

[64] Strosahl K (2005). Training behavioral health and primary care providers for integrated care: a core competencies approach. Behavioral Integrative Care: Treatments That Work in the Primary Care Setting.

[65] Schneeweiss S, Ahmed S, Burhan A, Campbell C Competency-based CPD: implications for physicians, CPD providers and health care institutions. Canada: Royal College of Physicians and Surgeons of Canada.

[66] Sargeant J, Bhanji F, Holmboe E, Kassen B, McFadyen R, Mazurek K Assessment and feedback for continuing competence and enhanced expertise in practice. Canada: Royal College of Physicians and Surgeons of Canada.

[67] Ghassemi M, Goldenberg A, Morris Q, Rudzicz F, Wang B, Zemel R (2019). Accessible data, health AI and the human right to benefit from science and its applications. Health Law Canada.

[68] O'Doherty D, Dromey M, Lougheed J, Hannigan A, Last J, McGrath D (2018). Barriers and solutions to online learning in medical education - an integrative review. BMC Med Educ.

[69] Park JY, Mills KA (2014). Enhancing interdisciplinary learning with a learning management system. MERLOT J Online Learn Teach.

[70] (2021). Global strategy on digital health 2020-2025. World Health Organization.

[71] Wiens J, Saria S, Sendak M, Ghassemi M, Liu VX, Doshi-Velez F, Jung K, Heller K, Kale D, Saeed M, Ossorio PN, Thadaney-Israni S, Goldenberg A (2019). Do no harm: a roadmap for responsible machine learning for health care. Nat Med.

[72] Westerman M, Teunissen P (2013). Oxford Textbook of Medical Education.

