# Supplementary material for: Artificial Intelligence Education Programs for Health Care Professionals: Scoping Review
Source: JMIR Med Educ. 2021 Dec 13;7(4):e31043. doi: 10.2196/31043 (PMC8713099; doi:10.2196/31043)
Supplement: Multimedia Appendix 1 [file mededu_v7i4e31043_app1.docx]

**Multimedia Appendix 1. Database search strategies.**

Searches run on July 7, 2020 in:

- Ovid Medline ALL
- Ovid Embase
- Ovid APA PsycINFO
- Ovid Emcare Nursing
- Ovid Cochrane Database of Systematic Reviews
- Ovid Cochrane Central Register of Controlled Trials
- EBSCO ERIC
- Clarivate Web of Science

Database(s): **Ovid MEDLINE(R) ALL**1946 to July 06, 2020
Search Strategy:

| **#** | **Searches** | **Results** |
| --- | --- | --- |
| 1 | exp Artificial Intelligence/ | 97195 |
| 2 | Pattern Recognition, Automated/ | 25214 |
| 3 | (artificial adj2 intelligence*).mp. | 29255 |
| 4 | big data.mp. | 7379 |
| 5 | (comput* adj3 intelligence*).mp. | 571 |
| 6 | (comput* adj2 knowledge adj2 representation*).mp. | 7 |
| 7 | data science.mp. | 1181 |
| 8 | (deep adj3 learn*).mp. | 11064 |
| 9 | (deep adj2 network*).mp. | 3897 |
| 10 | (machine adj3 intelligence*).mp. | 518 |
| 11 | (machine* adj3 learn*).mp. | 35199 |
| 12 | (ML adj2 framework).mp. | 57 |
| 13 | (ML adj2 tool*).mp. | 51 |
| 14 | ML algorithm*.mp. | 483 |
| 15 | ML application*.mp. | 117 |
| 16 | ML method*.mp. | 725 |
| 17 | ML model*.mp. | 460 |
| 18 | pattern classification.mp. | 1398 |
| 19 | (pattern recognition adj2 (comput* or automat* or artificial*)).mp. | 25449 |
| 20 | (data adj2 visuali?ation).mp. | 2453 |
| 21 | (information adj2 visuali?ation).mp. | 445 |
| 22 | (data adj2 literac*).mp. | 102 |
| 23 | (data adj2 literate*).mp. | 4 |
| 24 | or/1-23 | 149580 |
| 25 | exp *Schools, Health Occupations/ | 25780 |
| 26 | exp *Students, Health Occupations/ | 50013 |
| 27 | *education, predental/ or *education, premedical/ | 536 |
| 28 | exp *Health Occupations/ed or exp *Health Personnel/ed | 138794 |
| 29 | or/25-28 | 202316 |
| 30 | exp *Health Occupations/ or exp *Health Personnel/ | 1489126 |
| 31 | (nurs* or doctor* or physician* or therap* or psychiatrist* or psychologist* or surgeon* or social worker* or specialist* or "health care" or "healthcare" or medic* or hospital* or interdisciplin* or interprofession*).ti,kf,hw. | 4783853 |
| 32 | 30 or 31 | 5286413 |
| 33 | (student* or learner* or learned or learns or teach* or educat* or instruct* or class or classes or workshop? or module? or train??? or curricul* or upskill* or retrain??? or webinar* or elearn* or e-learn* or in-service or inservice or professional develop* or continuing professional or CPD or CME or life-long-learn* or lifelong* learn* or certificate program? or certification?).ti,kf,hw. | 1086195 |
| 34 | exp *education, professional/ | 216844 |
| 35 | exp *Teaching/ | 53380 |
| 36 | exp *Inservice Training/ | 14475 |
| 37 | exp *curriculum/ | 31929 |
| 38 | *professional competence/ | 11662 |
| 39 | *Education, distance/ | 3028 |
| 40 | (teach* or educat*).jw,in. | 577767 |
| 41 | or/33-40 | 1554472 |
| 42 | 32 and 41 | 654049 |
| 43 | 29 or 42 | 681620 |
| 44 | 24 and 43 | 2973 |

Database(s): **Embase**1974 to 2020 July 06
Search Strategy:

| **#** | **Searches** | **Results** |
| --- | --- | --- |
| 1 | exp artificial intelligence/ | 39681 |
| 2 | exp machine learning/ | 207418 |
| 3 | big data/ | 2130 |
| 4 | data science/ | 426 |
| 5 | (artificial adj2 intelligence*).mp. | 27614 |
| 6 | big data.mp. | 8939 |
| 7 | (comput* adj3 intelligence*).mp. | 787 |
| 8 | (comput* adj2 knowledge adj2 representation*).mp. | 12 |
| 9 | data science.mp. | 1223 |
| 10 | (deep adj3 learn*).mp. | 13982 |
| 11 | (deep adj2 network*).mp. | 4872 |
| 12 | (machine adj3 intelligence*).mp. | 619 |
| 13 | (machine* adj3 learn*).mp. | 49526 |
| 14 | (ML adj2 framework).mp. | 59 |
| 15 | (ML adj2 tool*).mp. | 77 |
| 16 | ML algorithm*.mp. | 660 |
| 17 | ML application*.mp. | 152 |
| 18 | ML method*.mp. | 1757 |
| 19 | ML model*.mp. | 628 |
| 20 | pattern classification.mp. | 1863 |
| 21 | (pattern recognition adj2 (comput* or automat* or artificial*)).mp. | 16723 |
| 22 | (data adj2 visuali?ation).mp. | 3020 |
| 23 | (information adj2 visuali?ation).mp. | 440 |
| 24 | (data adj2 literac*).mp. | 131 |
| 25 | (data adj2 literate*).mp. | 7 |
| 26 | or/1-25 | 240813 |
| 27 | *medical school/ or *pharmacy school/ | 16347 |
| 28 | exp *health student/ | 47383 |
| 29 | exp *health education/ or exp *medical education/ or exp *paramedical education/ | 317281 |
| 30 | or/27-29 | 349051 |
| 31 | *medical profession/ or *nursing as a profession/ or *nursing career/ or *paramedical profession/ or exp *health care personnel/ | 515213 |
| 32 | (nurs* or doctor* or physician* or therap* or psychiatrist* or psychologist* or surgeon* or social worker* or specialist* or "health care" or "healthcare" or medic* or hospital* or interdisciplin* or interprofession*).ti,kw,hw. | 8869261 |
| 33 | 31 or 32 | 8939480 |
| 34 | (student* or learner* or learned or learns or teach* or educat* or instruct* or class or classes or workshop? or module? or train??? or curricul* or upskill* or retrain??? or webinar* or elearn* or e-learn* or in-service or inservice or professional develop* or continuing professional or CPD or CME or life-long-learn* or lifelong* learn* or certificate program? or certification?).ti,kw,hw. | 1822285 |
| 35 | *continuing education/ or *course content/ or *education program/ or *in service training/ or exp *interdisciplinary education/ or *lifelong learning/ or *mentoring/ | 28642 |
| 36 | exp *Teaching/ | 36246 |
| 37 | *curriculum/ or *curriculum development/ | 24628 |
| 38 | *professional competence/ | 9990 |
| 39 | *professional development/ | 1897 |
| 40 | (teach* or educat*).jw,in. | 734711 |
| 41 | or/34-40 | 2390599 |
| 42 | 33 and 41 | 1205976 |
| 43 | 30 or 42 | 1286753 |
| 44 | 26 and 43 | 5042 |

Database(s): **APA PsycInfo**1806 to June Week 5 2020
Search Strategy:

| **#** | **Searches** | **Results** |
| --- | --- | --- |
| 1 | exp artificial intelligence/ | 21146 |
| 2 | big data/ | 908 |
| 3 | (artificial adj2 intelligence*).mp. | 9963 |
| 4 | big data.mp. | 1855 |
| 5 | (comput* adj3 intelligence*).mp. | 846 |
| 6 | (comput* adj2 knowledge adj2 representation*).mp. | 15 |
| 7 | data science.mp. | 215 |
| 8 | (deep adj3 learn*).mp. | 1840 |
| 9 | (deep adj2 network*).mp. | 700 |
| 10 | (machine adj3 intelligence*).mp. | 225 |
| 11 | (machine* adj3 learn*).mp. | 10607 |
| 12 | (ML adj2 framework).mp. | 10 |
| 13 | (ML adj2 tool*).mp. | 6 |
| 14 | ML algorithm*.mp. | 45 |
| 15 | ML application*.mp. | 3 |
| 16 | ML method*.mp. | 83 |
| 17 | ML model*.mp. | 49 |
| 18 | pattern classification.mp. | 673 |
| 19 | (pattern recognition adj2 (comput* or automat* or artificial*)).mp. | 898 |
| 20 | (data adj2 visuali?ation).mp. | 414 |
| 21 | (information adj2 visuali?ation).mp. | 201 |
| 22 | (data adj2 literac*).mp. | 290 |
| 23 | (data adj2 literate*).mp. | 18 |
| 24 | or/1-23 | 29573 |
| 25 | exp *medical education/ | 21667 |
| 26 | *nursing education/ | 5360 |
| 27 | *dental students/ or *medical students/ | 10677 |
| 28 | 25 or 26 or 27 | 32427 |
| 29 | exp *health personnel/ | 118650 |
| 30 | (nurs* or doctor* or physician* or therap* or psychiatrist* or psychologist* or surgeon* or social worker* or specialist* or "health care" or "healthcare" or medic* or hospital* or interdisciplin* or interprofession*).ti,id,hw. | 770758 |
| 31 | 29 or 30 | 806519 |
| 32 | exp *education/ | 358227 |
| 33 | exp *teaching/ | 101594 |
| 34 | (student* or learner* or learned or learns or teach* or educat* or instruct* or class or classes or workshop? or module? or train??? or curricul* or upskill* or retrain??? or webinar* or elearn* or e-learn* or in-service or inservice or professional develop* or continuing professional or CPD or CME or life-long-learn* or lifelong* learn* or certificate program? or certification?).ti,id,hw. | 875101 |
| 35 | 32 or 33 or 34 | 916506 |
| 36 | 31 and 35 | 129884 |
| 37 | 28 or 36 | 130929 |
| 38 | 24 and 37 | 261 |

Database(s): **Ovid Emcare Nursing**1995 to Present
Search Strategy:

| **#** | **Searches** | **Results** |
| --- | --- | --- |
| 1 | exp artificial intelligence/ | 8765 |
| 2 | exp machine learning/ | 44117 |
| 3 | big data/ | 640 |
| 4 | data science/ | 156 |
| 5 | (artificial adj2 intelligence*).mp. | 5186 |
| 6 | big data.mp. | 2919 |
| 7 | (comput* adj3 intelligence*).mp. | 216 |
| 8 | (comput* adj2 knowledge adj2 representation*).mp. | 12 |
| 9 | data science.mp. | 443 |
| 10 | (deep adj3 learn*).mp. | 3944 |
| 11 | (deep adj2 network*).mp. | 1180 |
| 12 | (machine adj3 intelligence*).mp. | 160 |
| 13 | (machine* adj3 learn*).mp. | 13923 |
| 14 | (ML adj2 framework).mp. | 20 |
| 15 | (ML adj2 tool*).mp. | 14 |
| 16 | ML algorithm*.mp. | 171 |
| 17 | ML application*.mp. | 22 |
| 18 | ML method*.mp. | 262 |
| 19 | ML model*.mp. | 130 |
| 20 | pattern classification.mp. | 537 |
| 21 | (pattern recognition adj2 (comput* or automat* or artificial*)).mp. | 401 |
| 22 | (data adj2 visuali?ation).mp. | 890 |
| 23 | (information adj2 visuali?ation).mp. | 189 |
| 24 | (data adj2 literac*).mp. | 81 |
| 25 | (data adj2 literate*).mp. | 4 |
| 26 | or/1-25 | 53172 |
| 27 | *medical school/ or *pharmacy school/ | 3992 |
| 28 | exp *health student/ | 23536 |
| 29 | exp *health education/ or exp *medical education/ or exp *paramedical education/ | 92293 |
| 30 | or/27-29 | 109407 |
| 31 | *medical profession/ or *nursing as a profession/ or *nursing career/ or *paramedical profession/ or exp *health care personnel/ | 208013 |
| 32 | (nurs* or doctor* or physician* or therap* or psychiatrist* or psychologist* or surgeon* or social worker* or specialist* or "health care" or "healthcare" or medic* or hospital* or interdisciplin* or interprofession*).ti,kw,hw. | 2489251 |
| 33 | 31 or 32 | 2509599 |
| 34 | (student* or learner* or learned or learns or teach* or educat* or instruct* or class or classes or workshop? or module? or train??? or curricul* or upskill* or retrain??? or webinar* or elearn* or e-learn* or in-service or inservice or professional develop* or continuing professional or CPD or CME or life-long-learn* or lifelong* learn* or certificate program? or certification?).ti,kw,hw. | 731866 |
| 35 | *continuing education/ or *course content/ or *education program/ or *in service training/ or exp *interdisciplinary education/ or *lifelong learning/ or *mentoring/ | 10896 |
| 36 | exp *Teaching/ | 16025 |
| 37 | *curriculum/ or *curriculum development/ | 11159 |
| 38 | *professional competence/ | 1977 |
| 39 | *professional development/ | 3527 |
| 40 | (teach* or educat*).jw,in. | 308795 |
| 41 | or/34-40 | 923555 |
| 42 | 33 and 41 | 488885 |
| 43 | 30 or 42 | 515841 |
| 44 | 26 and 43 | 2127 |

Database(s): **Cochrane Database of Systematic Reviews**2005 to Present
Search Strategy:

| **#** | **Searches** | **Results** |
| --- | --- | --- |
| 1 | (artificial adj2 intelligence*).mp. | 28 |
| 2 | big data.mp. | 8 |
| 3 | (comput* adj3 intelligence*).mp. | 3 |
| 4 | (comput* adj2 knowledge adj2 representation*).mp. | 0 |
| 5 | data science.mp. | 1 |
| 6 | (deep adj3 learn*).mp. | 3 |
| 7 | (deep adj2 network*).mp. | 0 |
| 8 | (machine adj3 intelligence*).mp. | 1 |
| 9 | (machine* adj3 learn*).mp. | 41 |
| 10 | (ML adj2 framework).mp. | 0 |
| 11 | (ML adj2 tool*).mp. | 0 |
| 12 | ML algorithm*.mp. | 0 |
| 13 | ML application*.mp. | 2 |
| 14 | ML method*.mp. | 0 |
| 15 | ML model*.mp. | 0 |
| 16 | pattern classification.mp. | 1 |
| 17 | (pattern recognition adj2 (comput* or automat* or artificial*)).mp. | 0 |
| 18 | (data adj2 visuali?ation).mp. | 3 |
| 19 | (information adj2 visuali?ation).mp. | 1 |
| 20 | (data adj2 literac*).mp. | 0 |
| 21 | (data adj2 literate*).mp. | 0 |
| 22 | or/1-21 | 79 |

Database(s): **Cochrane Central Register of Controlled Trials**2014 to Present
Search Strategy:

| **#** | **Searches** | **Results** |
| --- | --- | --- |
| 1 | exp Artificial Intelligence/ or exp Artificial Intelligence/ | 958 |
| 2 | Pattern Recognition, Automated/ | 220 |
| 3 | (artificial adj2 intelligence*).mp. | 452 |
| 4 | big data/ or big data.mp. | 132 |
| 5 | (comput* adj3 intelligence*).mp. | 37 |
| 6 | (comput* adj2 knowledge adj2 representation*).mp. | 0 |
| 7 | data science/ or data science.mp. | 18 |
| 8 | (deep adj3 learn*).mp. | 333 |
| 9 | (deep adj2 network*).mp. | 97 |
| 10 | (machine adj3 intelligence*).mp. | 17 |
| 11 | (machine* adj3 learn*).mp. | 1147 |
| 12 | (ML adj2 framework).mp. | 1 |
| 13 | (ML adj2 tool*).mp. | 11 |
| 14 | ML algorithm*.mp. | 41 |
| 15 | ML application*.mp. | 22 |
| 16 | ML method*.mp. | 168 |
| 17 | ML model*.mp. | 42 |
| 18 | pattern classification.mp. | 47 |
| 19 | (pattern recognition adj2 (comput* or automat* or artificial*)).mp. | 236 |
| 20 | (data adj2 visuali?ation).mp. | 66 |
| 21 | (information adj2 visuali?ation).mp. | 15 |
| 22 | (data adj2 literac*).mp. | 21 |
| 23 | (data adj2 literate*).mp. | 0 |
| 24 | or/1-23 | 3125 |
| 25 | exp *Schools, Health Occupations/ or *medical school/ or *pharmacy school/ | 7 |
| 26 | exp *Students, Health Occupations/ or exp *health student/ | 581 |
| 27 | *education, predental/ or *education, premedical/ or exp *health education/ or exp *Education, Medical/ or exp *paramedical education/ | 6773 |
| 28 | exp *Health Occupations/ed or exp *Health Personnel/ed | 214 |
| 29 | or/25-28 | 7389 |
| 30 | exp *Health Occupations/ or exp *Health Personnel/ or *medical profession/ or *nursing as a profession/ or *nursing career/ or *paramedical profession/ or exp *health care personnel/ | 10146 |
| 31 | (nurs* or doctor* or physician* or therap* or psychiatrist* or psychologist* or surgeon* or social worker* or specialist* or "health care" or "healthcare" or medic* or hospital* or interdisciplin* or interprofession*).ti,kw,hw. | 593419 |
| 32 | 30 or 31 | 596062 |
| 33 | (student* or learner* or learned or learns or teach* or educat* or instruct* or class or classes or workshop? or module? or train??? or curricul* or upskill* or retrain??? or webinar* or elearn* or e-learn* or in-service or inservice or professional develop* or continuing professional or CPD or CME or life-long-learn* or lifelong* learn* or certificate program? or certification?).ti,kw,hw. | 128733 |
| 34 | exp *education, professional/ or *continuing education/ or *course content/ or *education program/ or *in service training/ or exp *interdisciplinary education/ or *lifelong learning/ or *mentoring/ | 2254 |
| 35 | exp *Teaching/ | 1428 |
| 36 | exp *Inservice Training/ | 0 |
| 37 | exp *curriculum/ or *curriculum development/ | 2 |
| 38 | *professional competence/ | 0 |
| 39 | *Education, distance/ | 2 |
| 40 | *professional development/ | 0 |
| 41 | (teach* or educat*).jw,in. | 14805 |
| 42 | or/33-41 | 137865 |
| 43 | 32 and 42 | 68348 |
| 44 | 29 or 43 | 70897 |
| 45 | 24 and 44 | 335 |

**Database - ERIC**

Interface - EBSCOhost Research Databases
Tuesday, July 07, 2020 2:03:13 PM

| **#** | **Query** | **Results** |
| --- | --- | --- |
| S29 | S25 AND S28 | 2,035 |
| S28 | S26 OR S27 | 175,300 |
| S27 | (nurs* or doctor* or physician* or therapist* or psychiatrist* or psychologist* or surgeon* or social worker* or specialist* or "health care" or "healthcare" or medic* or hospital* or interdisciplin* or interprofession*) | 170,480 |
| S26 | DE "Medical Schools" OR DE "Dental Schools" OR DE "Foreign Medical Graduates" OR DE "Graduate Medical Education" OR DE "Medical Education" OR DE "Medical School Faculty" OR DE "Medical Students" OR DE "Premedical Students" OR DE "Nursing Education" OR DE "Pharmaceutical Education" OR DE "Veterinary Medical Education" OR DE "Allied Health Occupations Education" OR DE "Clinical Teaching (Health Professions)" OR DE "Health Occupations" OR DE "Allied Health Occupations" OR DE "Health Personnel" OR DE "Allied Health Personnel" OR DE "Mental Health Workers" OR DE "Nurses" OR DE "Physicians" OR DE "Psychologists" | 35,692 |
| S25 | S1 OR S2 OR S3 OR S4 OR S5 OR S6 OR S7 OR S8 OR S9 OR S10 OR S11 OR S12 OR S13 OR S14 OR S15 OR S16 OR S17 OR S18 OR S19 OR S20 OR S21 OR S22 OR S23 OR S24 | 24,493 |
| S24 | data N2 literate* | 27 |
| S23 | data N2 literac* | 624 |
| S22 | data N2 visualization | 255 |
| S21 | data N2 visualisation | 255 |
| S20 | pattern recognition N2 artificial* | 1 |
| S19 | pattern recognition N2 automat* | 7 |
| S18 | pattern recognition N2 comput* | 10 |
| S17 | pattern classification | 31 |
| S16 | ML model* | 6 |
| S15 | ML method* | 10 |
| S14 | ML application* | 0 |
| S13 | ML algorithm* | 3 |
| S12 | ML N2 tool* | 4 |
| S11 | ML N2 framework | 2 |
| S10 | machine* N3 learn* | 707 |
| S9 | machine* N3 intelligence* | 50 |
| S8 | deep N2 network* | 17 |
| S7 | deep N3 learn* | 1,382 |
| S6 | data science | 115 |
| S5 | comput* N2 knowledge N2 representation* | 9 |
| S4 | comput* N3 intelligence* | 207 |
| S3 | big data | 19,416 |
| S2 | artificial N2 intelligence* | 2,467 |
| S1 | DE "Artificial Intelligence" | 1,953 |

| **Web of Science**  July 7, 2020  Indexes=SCI-EXPANDED, SSCI, A&HCI, CPCI-S, CPCI-SSH, BKCI-S, BKCI-SSH, ESCI  Timespan=All years | | |
| --- | --- | --- |
| Set | Results | Save History / Create AlertOpen Saved History |
| # 11 | 597 | #10 AND #9 |
| # 10 | 46944 | TI=((artificial NEAR2 intelligence) OR big data OR (comput* NEAR3 intelligence) OR data science OR (comput* NEAR2 knowledge NEAR2 representation*) OR (deep NEAR3 learn*) OR (deep NEAR2 network*) OR (machine NEAR3 intelligence*) OR (machine NEAR3 learn*) OR (ML NEAR2 framework) OR (ML NEAR2 tool*) OR ML algorithm* OR ML application* OR ML method* OR ML model* OR pattern classification OR (pattern recognition NEAR2 comput*) OR (pattern recognition NEAR2 automat*) OR (pattern recognition NEAR2 artificial*) OR (data NEAR2 visualisation) OR (data NEAR2 visualization) OR (data NEAR2 literac*) OR (data NEAR2 literate*) ) OR KP=((artificial NEAR2 intelligence) OR big data OR (comput* NEAR3 intelligence) OR data science OR (comput* NEAR2 knowledge NEAR2 representation*) OR (deep NEAR3 learn*) OR (deep NEAR2 network*) OR (machine NEAR3 intelligence*) OR (machine NEAR3 learn*) OR (ML NEAR2 framework) OR (ML NEAR2 tool*) OR ML algorithm* OR ML application* OR ML method* OR ML model* OR pattern classification OR (pattern recognition NEAR2 comput*) OR (pattern recognition NEAR2 automat*) OR (pattern recognition NEAR2 artificial*) OR (data NEAR2 visualisation) OR (data NEAR2 visualization) OR (data NEAR2 literac*) OR (data NEAR2 literate*) ) |
| # 9 | 496527 | #8 AND #4 |
| # 8 | 3096144 | #7 OR #6 OR #5 |
| # 7 | 2437610 | TI=(student* or learner* or learned or learns or teach* or educat* or instruct* or class or classes or workshop* or module* or train* or curricul* or upskill* or retrain* or webinar* or elearn* or e-learn* or in-service or inservice or professional develop* or continuing professional or CPD or CME or life-long-learn* or lifelong* learn* or certificate program? or certification?) OR KP=(student* or learner* or learned or learns or teach* or educat* or instruct* or class or classes or workshop* or module* or train* or curricul* or upskill* or retrain* or webinar* or elearn* or e-learn* or in-service or inservice or professional develop* or continuing professional or CPD or CME or life-long-learn* or lifelong* learn* or certificate program? or certification?) |
| # 6 | 1343976 | WC=(educat*) |
| # 5 | 1254012 | SU=(Education & Educational Research) |
| # 4 | 11506336 | #3 OR #2 OR #1 |
| # 3 | 1751597 | TI=(nurs* or doctor* or physician* or therapist* or psychiatrist* or psychologist* or surgeon* or social worker* or specialist* or "health care" or "healthcare" or medic* or hospital* or interdisciplin* or interprofession*) OR KP=(nurs* or doctor* or physician* or therapist* or psychiatrist* or psychologist* or surgeon* or social worker* or specialist* or "health care" or "healthcare" or medic* or hospital* or interdisciplin* or interprofession*) |
| # 2 | 959163 | SU=(Life Sciences & Biomedicine) |
| # 1 | 9762983 | WC=(medical* or medicine* or health* or nursing* or clinical* or biomed*) |
